# Effects of experimental nest treatment with herbs on ectoparasites and body condition of nestlings

**DOI:** 10.1093/beheco/arae103

**Published:** 2024-12-13

**Authors:** Michał Glądalski, Ana Cláudia Norte, Maciej Bartos, Iwona Demeško, Adam Kaliński, Marcin Markowski, Joanna Skwarska, Jarosław Wawrzyniak, Piotr Zieliński, Jerzy Bańbura

**Affiliations:** MARE–Marine and Environmental Sciences Centre, Department of Life Sciences, University of Coimbra, Coimbra, 3000-456, Portugal; Faculty of Biology and Environmental Protection, Department of Experimental Zoology and Evolutionary Biology, University of Lodz, Banacha 12/16, 90-237 Lodz, Poland; MARE–Marine and Environmental Sciences Centre, Department of Life Sciences, University of Coimbra, Coimbra, 3000-456, Portugal; Faculty of Biology and Environmental Protection, Department of Biodiversity Studies and Bioeducation, University of Lodz, Banacha 1/3, 90-237 Lodz, Poland; Faculty of Biology and Environmental Protection, Department of Experimental Zoology and Evolutionary Biology, University of Lodz, Banacha 12/16, 90-237 Lodz, Poland; Faculty of Biology and Environmental Protection, Department of Experimental Zoology and Evolutionary Biology, University of Lodz, Banacha 12/16, 90-237 Lodz, Poland; Faculty of Biology and Environmental Protection, Department of Experimental Zoology and Evolutionary Biology, University of Lodz, Banacha 12/16, 90-237 Lodz, Poland; Faculty of Biology and Environmental Protection, Department of Experimental Zoology and Evolutionary Biology, University of Lodz, Banacha 12/16, 90-237 Lodz, Poland; Faculty of Biology and Environmental Protection, Department of Experimental Zoology and Evolutionary Biology, University of Lodz, Banacha 12/16, 90-237 Lodz, Poland; Faculty of Biology and Environmental Protection, Department of Ecology and Vertebrate Zoology, University of Lodz, Banacha 12/16, 90-237 Lodz, Poland; Faculty of Biology and Environmental Protection, Department of Experimental Zoology and Evolutionary Biology, University of Lodz, Banacha 12/16, 90-237 Lodz, Poland

**Keywords:** aromatic herbs, Avian nests, drug hypothesis, nest protection hypothesis, hemoglobin, hematocrit, hole-nesting birds, parasite abundance, ticks

## Abstract

Nest fumigation behavior involves the incorporation of fresh green plant fragments that contain ectoparasite-repellent volatile compounds into birds’ nests. This behavior is relatively rare among bird species, and there is ongoing debate about whether it benefits parental breeding success. In this study, we experimentally tested whether the inclusion of aromatic-herbal plant fragments in the nests of great tits *Parus major* affects the physiological condition of nestlings, as indicated by blood levels of hematocrit, hemoglobin, glucose, and body condition indices, such as weight and wing length. We divided the nests into 2 groups, adding aromatic herbs to the test group’s nests and non-aromatic plants to the control group. After the nestlings fledged, all nest materials were collected to extract, identify, and count arthropod ectoparasites. Nestlings in nests supplemented with aromatic plant fragments had elevated levels of hematocrit and hemoglobin, indicating improved physiological condition compared to the control group. Ectoparasites were present in both groups, although ticks (Ixodidae) occurred less frequently in nests with aromatic plants. The experimental treatment did not affect fledging success. Further experimental studies are needed to explore the effects of incorporating aromatic plant fragments into tit nests within the frameworks of both the nest protection hypothesis and the drug hypothesis.

## Teaser

We experimentally tested whether the inclusion of aromatic-herbal plant fragments in the nests of great tits (*Parus major*) affects the condition of nestlings and arthropod numbers in the nests. Ectoparasites were present in both experimental and control groups, although ticks (Ixodidae) occurred less frequently in nests with aromatic plants. The experimental treatment did not affect fledging success.

## Introduction

Birds have been shown to employ several behavioral mechanisms to repel or combat parasites. Among these strategies are migration, avoidance of parasitized prey, tolerance, body maintenance behaviors (such as bathing, dust bathing, and preening), nest maintenance behaviors (including nest sanitation), and nest fumigation. Nest fumigation involves incorporating fresh herb fragments that produce volatile compounds into the nests (West et al. 2015; Bush and Clayton 2018; Deeming 2023; Lambrechts and Deeming 2024). Several species of hole-nesting passerines, such as European starlings *Sturnus vulgaris*, tree swallows *Tachycineta bicolor*, russet sparrows *Passer cinnamomeus*, and both blue *Cyanistes caeruleus* and great tits, have been observed incorporating fresh, aromatic plant fragments into their nests (Dubiec et al. 2013; Tomás et al. 2013; Mainwaring 2017; Gwinner et al. 2018; Yang et al. 2020; Mainwaring et al. 2023). Petit et al. (2002) demonstrated that, out of more than 200 plant species available in their study area in Corsica, only about 10 species were brought by blue tit females to their nests. It is also important to note that, according to Lambrechts and Dos Santos (2000), herb fragments may dry out quickly and become unrecognizable in collected nests. Consequently, in Petit’s study, about 10 herb species were frequently delivered to nests and identified, though the actual number of herb species used could potentially be higher.

There are 2 main hypotheses that may explain the behavior of bringing herbs to nests. The “nest protection hypothesis” suggests that some birds may add aromatic plants to their nests to repel or kill ectoparasites (Wimberger 1984; Pires et al. 2012). Several aromatic, volatile compound-producing plants used by these bird species, such as *Lavandula angustifolia*, *Achillea millefolium*, *Mentha* spp., *Helichrysum italicum*, *Glechoma hederacea*, and *Artemisia verlotorum*, have been shown to have repellent effects against blood-sucking ectoparasites and other insects (Adam et al. 1998; Lafuma et al. 2001; Maia and Moore 2011; Kamatou et al. 2013; De Almeida et al. 2017; Germinara et al. 2017; Stenning 2018; Garrido-Bautista et al. 2023). The second hypothesis, the “drug hypothesis,” proposes that aromatic plant fragments in avian nests may have direct beneficial effects on nestling condition and development by enhancing immune function (Gwinner et al. 2000; Pires et al. 2020). It is also possible that these 2 hypotheses are not mutually exclusive. In contrast to the many studies focusing on incubation, brooding, and chick feeding in secondary hole-nesting birds, surprisingly few have explored hygienic behaviors such as nest sanitation and fumigation as components of parental care (Bańbura et al. 2001; Mennerat et al. 2008, 2009b, Glądalski et al. 2020; Pires et al. 2020; Garrido-Bautista et al. 2023). Even fewer studies have examined the relationship between aromatic plant fragments in tit nests and body condition indices (Dubiec et al. 2013; Scott-Baumann and Morgan 2015), and the limited available results remain inconclusive (Mainwaring et al. 2014; Scott-Baumann and Morgan 2015; Pires et al. 2020; Garrido-Bautista et al. 2023). Due to the contradictory findings regarding the effects of aromatic plants on ectoparasite pressure and nestling health (whether directly or indirectly mediated by ectoparasites), further experimental studies are necessary to assess the potential benefits of this behavior. In Parids, experimental manipulations of aromatic plant content in nests have so far been conducted exclusively with blue tits (Petit et al. 2002; Mennerat et al. 2008, 2009a, b; Tomás et al. 2012; Glądalski et al. 2020; Pires et al. 2020; Scott-Baumann et al. 2022; Garrido-Bautista et al. 2023), while no such studies have been conducted with great tits.

Nests of secondary hole-nesting birds are characterized by a moist and warm environment, rich in organic matter from leftover nestling food, feces, and organic powder (a by-product of feather growth) (Britt and Deeming 2011; Harnist et al. 2020). This environment creates an ideal microhabitat for a wide variety of arthropods, bacteria, fungi, and other opportunistic organisms (López-Rull and Macías Garcia 2015; Mainwaring 2016, 2017; Hanmer et al. 2017; Zabłotni et al. 2020, 2023). Most ectoparasites found in avian nests are insects and arachnids that feed on the blood, skin, organic powder, feathers, or dead nestlings of both nestlings and parents (Bańbura et al. 1995, 2001; Reynolds et al. 2016). Nest-dwelling parasites have significant potential to affect the life histories of both nestlings and parents, often exerting selective pressure (Hamilton and Zuk 1982). Blood-sucking ectoparasites, such as fleas (Ceratophyllidae), blow flies (Calliphoridae), ticks (Ixodida), and parasitic mites (Parasitiformes and Acariformes), are particularly harmful to their hosts, draining resources and causing damage that can range from subtle to severe, or even lethal effects (López-Rull and Macías Garcia 2015; see also Gallizzi and Richner 2008). Body condition is considered a key factor affecting the fitness of wild animals. It can be assessed through various types of morphological and/or physiological measurements (Brown 1996; Minias 2015; Norte et al. 2022). For instance, body mass, wing length, and fat scores are morphometric indicators, while other condition indices are based on biochemical or physiological parameters (e.g. plasma-lipid metabolites, hormone levels, hematological parameters). Body mass, wing length, and hematological markers (e.g. hemoglobin, glucose, and hematocrit levels) are commonly used in ornithological studies of both adult birds and chicks, as they are sensitive indicators and early warnings of environmental changes (Nadolski et al. 2006; Norte et al. 2008; Kaliński et al. 2011, 2014, 2022; Labocha and Hayes 2012; Johnstone et al. 2015; Markowski et al. 2015; Minias 2015). Hemoglobin concentration is regarded as a reliable indicator of body condition and is positively associated with nestling health (Johnstone et al. 2015; Kaliński et al. 2015a; Glądalski et al. 2018a, 2020). Variations in hemoglobin levels can be caused by several factors, including pressure from blood-sucking parasites (Minias 2015). Blood glucose concentration is largely reflective of a bird’s metabolic rate (Minias 2014; Glądalski et al. 2015, 2018a; Kaliński et al. 2015b, 2016); however, due to its high variability, it is considered a less robust indicator of health, with stress leading to elevated values (Lill 2011; Kaliński et al. 2014). Hematocrit, a measure of the compacted volume of red blood cells relative to total blood volume, has been described in several studies as a useful indicator of a bird’s physiological or body condition (Potti et al. 1999; Fair et al. 2007; Markowski et al. 2015, 2019). A reduction in hematocrit indicates anemia, which in passerines typically results from blood loss caused by blood-sucking parasites (Potti et al. 1999; Fair et al. 2007; Markowski et al. 2019). In blue tit nestlings, Simon et al. (2005) found that changes in hematocrit levels depend almost entirely on parasite loads in the nest. Similarly, Potti et al. (1999) demonstrated that hematocrit levels in Pied Flycatcher *Ficedula hypoleuca* nestlings are significantly affected by blood-sucking ectoparasitic mites.Minias 2014; Glądalski et al. 2015; Kaliński et al. 2015b, 2016; Glądalski et al. 2018a

The great tit is a sister species to the blue tit, with similar breeding ecology, and is also affected by ectoparasites in closed nests (Scott-Baumann et al. 2022). We would expect the advantages of herb use to be similar for both species. Our proposed field experiment adopts a medicine-based approach, where “drugs” can be tested outside a natural coevolutionary framework. The underlying assumption is that physiological responses to aromatic plants will not depend on whether the model species frequently use herbs in natural conditions. Therefore, in this study, we aimed to experimentally test whether the supplementation of fresh aromatic plants in great tit nests affects indices of nestling physiological and body condition (and thus nestling health), ectoparasite abundance, and fledging success in a deciduous forest in central Poland. Our hypothesis is that great tit nestlings from nests with added fresh aromatic plants should exhibit higher concentrations of hemoglobin and hematocrit, lower glucose concentrations, greater body mass, and longer wings compared to nestlings in the control group. In addition, nests with added fresh aromatic plants should have reduced ectoparasite abundance compared to the control group.

## Materials and methods

### Study areas

This study, carried out in 2023, is part of a long-term research project investigating the breeding biology of secondary cavity nesters around the city of Łódź, central Poland (Glądalski et al. 2018b, 2024). The study area (51°50ʹN, 19°29ʹE), bordering the north-east part of Łódź, is a forest site 140 ha in the center of a mature mixed deciduous Łagiewniki forest (about 1,250 ha in total) with 2 oak species: sessile oak *Quercus petraea* and pedunculate oak *Quercus robur* as predominating tree species (> 50% of all tree species), including also maples *Acer sp.,* hornbeam *Carpinus betulus*, birches *Betula pendula*, limes *Tilia sp*. The study area was supplied with ~330 standard wooden nestboxes with removable front wall (Lambrechts et al. 2010). The average distance between neighboring nestboxes was about 50 m. All nestboxes were cleaned out prior to the breeding season using a hand scrubber and handheld vacuum cleaner (BOSCH UniversalVac 06033B9103).

### Field and experimental procedures

During the breeding season, nestboxes were visited every day to record the laying date and the number hatchlings. From 26 nests of great tits with incubated clutches and without fresh plant material supplemented by birds on their own, we established pairs of nests according to their hatching date and brood size (± 2 nestlings). Thirteen nests were randomly assigned to the experimental treatment and 13 were control broods. Three nests from the experimental treatment were lost (when the nestlings were 5 to 8 d old) due to predation. The mean laying date (50.70 ± 1.42 SD vs. 50.76 ± 1.59 SD days from 1st March; *t* = 0.11, df = 21, *P* = 0.92), mean clutch size (9.40 ± 0.84 SD vs. 9.54 ± 1.13 SD eggs per nest; *t* = 0.32, df = 21, *P* = 0.75), and the mean number of nestlings on the 13th day after hatching (8.40 ± 2.07 SD vs. 8.69 ± 1.18 SD nestlings per nest; *t* = 0.43, df = 21, *P* = 0.67) did not differ between test and control groups.

Starting on day 2 and then every other day until day 16 after hatching, a 1.5-g portion of fresh aromatic plant fragments (~0.45 g of lavender *Lavandula angustifolia*, 0.35 g of mint *Mentha sp*., 0.35 g of the Immortelle *Helichrysum italicum*, and 0.35 g of the Common Yarrow *Achillea millefolium)* was supplemented in test nests and a 1.5-g portion of fresh grass *Poaceae spp.* was added to control nests. Both aromatic herbs and grass were placed on 3 edges of the lining layer of the nest (near back wall, left wall, and right wall). All plant species used in the experiment normally occur in the geographical distribution of the great tit and are found in natural nests of blue and great tits (Petit et al. 2002; Dubiec et al. 2013, Mainwaring 2017). These aromatic plants were not available in the bird territories or in the forest study area. At our study sites, only some blue tits and very occasionally use *Lamium galeobdolon* and *Veronica sp*. Unlike blue tits, great tits will not introduce new herbs during the experiment, allowing for better control of experimental conditions. All the aromatic herb species used were acquired in garden stores about 2 wk before the experiment and were grown in appropriate conditions until the end of the experimental procedures. The grass was obtained from the faculty botanic garden. In the morning, before visiting the study site, fresh plant fragments were cut off the aromatic plants, weighed, and put into plastic pouches, and a similar treatment was given to the grass.

### Body condition and physiological measurements

On day 13th after hatching, the nestlings were banded with individually numbered aluminum rings. A random sample of 3 nestlings from each brood was assigned for measurements: weighting (using a portable weighing scale) to the nearest 0.1 g, measuring (using an ornithological ruler) the length of the wing to the nearest 1 mm (with a standard protocol which is measuring the closed wing from the carpal joint to the tip of the longest primary feather), and blood sampling (in total 67 nestlings were blood sampled). Blood samples of ~5 μL from the ulnar vein were taken by venipuncture using a sterile needle. Blood was collected into HemoCue cuvettes and immediately analyzed in the field using a portable HemoCue Hb 201 + and HemoCue Glucose 201 + photometers (HemoCue AB, Angelholm, Sweden) to measure hemoglobin (g/l) and glucose (mg/dL) concentrations, respectively. Additional blood (~19 μL) was collected into heparinized microcapillary tubes. These blood samples were transported to the laboratory in a portable cooler and stored in a refrigerator until processing (later on the same day) to determine the hematocrit after centrifugation at 12,000 revolutions per minute for 10 min (Kaliński et al. 2011; Markowski et al. 2015). Hematocrit (%) was obtained by measuring the length of capillary occupied by packed red blood cells in relation to the total length of capillary filled with the blood sample. The measurements were performed using a caliper (to the nearest 0.1 mm). All field procedures were performed between 9:00 and 14:00 h.

### Analysis of nest material

Soon after the last nestling had left the nestbox the whole nest material was collected. Nest material was placed in a plastic bag, and the remaining nesting material in the bottom of the nestbox and walls, including invertebrates, was collected using a handheld vacuum cleaner (BOSCH UniversalVac 06033B9103). One nest was lost during collection procedures. The contents of the nest were poisoned using ethyl acetate and frozen at −18 °C. After thawing, the contents were sieved through a set of meshes (mesh sizes: 5, 0.5, 0.3, and 0.125 mm). Each nest was divided into 4 equal parts, of which one, a randomly selected part, was examined visually in detail by naked eye to extract all arthropod ectoparasites which were identified under an optical microscope to the family level.

### Statistical analyses

Because the values of hemoglobin, hematocrit, and glucose are not independent, we started modeling from extracting 2 principal components from their correlation matrix to get a joint multivariate indices of hemoglobin/hematocrit (PC1) and glucose (PC2) to control the data for the lack of independence (Manly and Navarro Alberto 2017). The PC1 index explained 60% (eigenvalue 1.80) and PC2 explained 25% (eigenvalue 0.76) of the joint variation in hemoglobin, hematocrit, and glucose. The PC1 was positively correlated with the values of hemoglobin concentration and hematocrit (loadings 0.79 and 0.86, respectively) and negatively correlated with glucose concentration (loading −0.67). The PC2 was strongly correlated with glucose concentration (loading 0.72) and relatively weakly correlated with hemoglobin and hematocrit (loadings 0.47 and 0.36, respectively). The PC indices, as characterized by mutual independence, were then modeled in separate models as the dependent variables in relation to the experimental factor; afterward, hemoglobin, hematocrit, and glucose were analyzed in the same way to check for the way in which the experiment influenced them.

The PC indices as well as the values of hemoglobin and glucose concentration in the blood, hematocrit, body mass, and wing length of nestlings from the same brood were not independent. Therefore, the individual nestling values (as unit records) were analyzed using mixed linear models, with the brood ID being included as a random effect (to control for clustering) and with degrees of freedom being approximated by the Satterthwaite method (Heck et al. 2010). Experimental treatment was treated as a fixed factor in these models. Linear mixed modeling was conducted using IBM SPSS Statistics 22 software (Heck et al. 2012; IBM SPSS 22, 2013). We checked for an effect of the experimental treatments on fledging success (binomial variable: a proportion of hatchlings that fledged per brood) using a binomial generalized linear model (Crawley 2002). *T*-tests and Mann–Whitney *U*-tests were also conducted. We considered *P* ≤ 0.05 as significant.

## Results

**Fig. 1.**
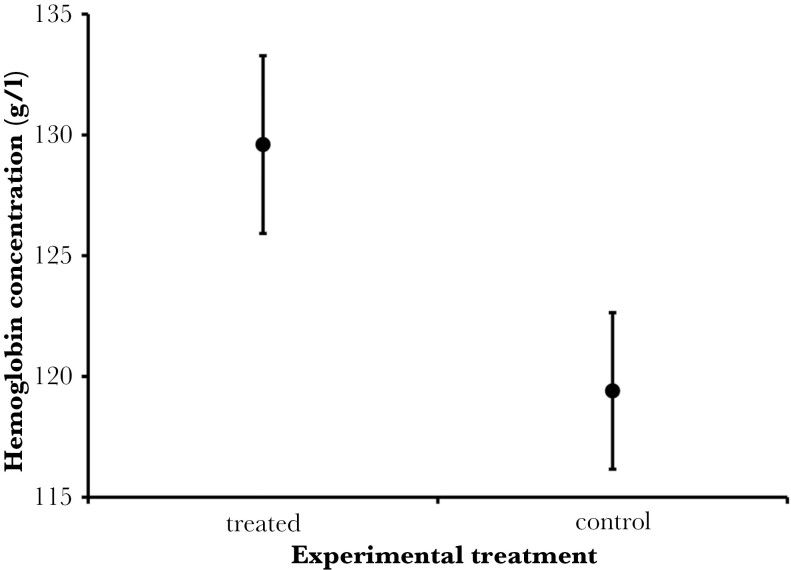
Mean hemoglobin concentration (g/l) in the blood of great tit *parus major* nestlings in experimentally tested group with aromatic plant fragments (129.6 ± 3.7 g/l) and control group (119.7 ± 3.2 g/l). Data are shown as mean ± SE.

**Fig. 2.**
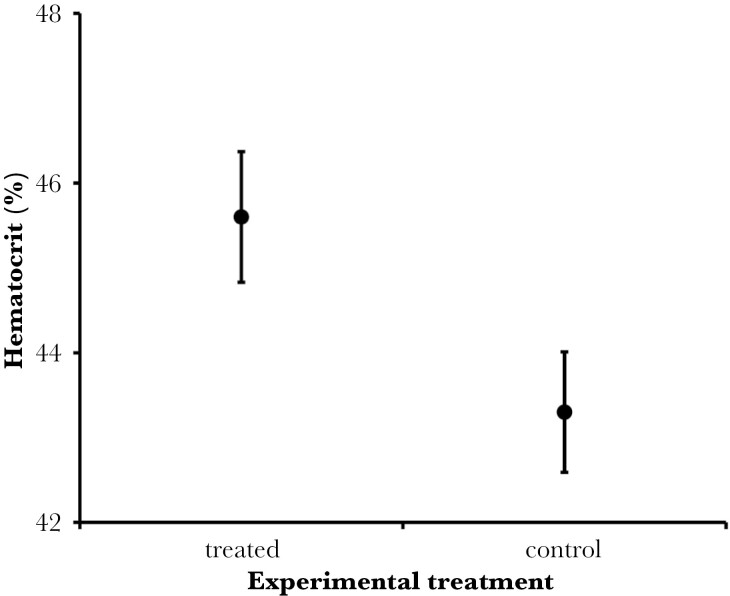
Mean hematocrit values (%) in the blood of the great tit Parus major nestlings in experimentally tested group with aromatic plant fragments placed (45.6 ± 0.8%) and control group (43.3 ± 0.7%). Data are shown as mean ± SE.

A total of 23 great tit broods were included in the present study (67 nestlings were bled). The first principal component, PC1—indicating levels of hemoglobin and hematocrit, was significantly higher in herb-treated nests than in control nests, whereas there was no effect of the experiment on the second principal component, PC2—indicating mostly glucose concentration (Table 1). Per-nest mean levels of hemoglobin ranged from 101.7 g/l to 149.0 g/l. The nestlings from the group with added aromatic plants had a significantly higher hemoglobin concentration (on average 10.2 g/l higher) than the nestlings from the control group (Table 1, Fig. 1). Per-nest mean levels of glucose varied from 199.7 mg/dl to 318.0 mg/dl. The concentration of glucose did not differ between the test and control groups (Table 1). Per-nest mean values of hematocrit ranged from 40.0% to 49.2%. Mean hematocrit values were higher in the test group (on average 2.3%) compared to control (Table 1, Fig. 2). Both body mass and wing length, did not show any differences between test and control groups (Table 1). Per-nest mass values ranged from 10.9 g to 17.8 g and per-nest wing length values ranged from 39.0 mm to 51.0 mm (Table 2). The experimental treatment did not influence fledging success (GLZM: χ^2^_1_ = 1.403, *P* = 0.236), which was 0.94 ± 0.022 (SE) in control broods and 0.98 ± 0.016 (SE) in treated broods.

**Table 1.** Linear mixed model analysis of the effects of aromatic plant experimental addition on PC1 (hemoglobin and hematocrit related) and PC2 (glucose related) but also blood hemoglobin and glucose concentrations, hematocrit, body mass and wing length of great tit *Parus major* nestlings. Significant values are in bold.

Factor	*Df*	*F*	*P*
**PC1 (Hb/Hct)**			
Intercept	1; 21.1	0.6	0,808
Treatment	1; 21.1	6.7	**0.017**
**PC2 (Glu)**			
Intercept	1; 20.0	0.1	0.960
Treatment	1; 20.0	0.1	0.714
**Hemoglobin**			
Intercept	1; 21.1	2583.1	**<0.001**
Treatment	1; 21.1	4.3	**0.050**
**Glucose**			
Intercept	1; 18.1	2324.6	**<0.001**
Treatment	1; 18.1	3.1	0.094
**Hematocrit**			
Intercept	1; 20.2	7211.6	**<0.001**
Treatment	1; 20.2	4.5	**0.047**
**Body mass**			
Intercept	1; 19.8	3164.2	**<0.001**
Treatment	1; 19.8	0.6	0.453
**Wing length**			
Intercept	1; 20.9	4232.4	**<0.001**
Treatment	1; 20.9	1.9	0.185

**Table 2.** Reproductive parameters, physiological measures, and body condition in great tit *Parus major* nestlings subjected to fresh aromatic plants addition to their nests (treatment) and in control nests.

	Treated			Control		
	Mean	SD	*N*	Mean	SD	*N*
Brood level						
Laying dates (f. 1st March)	50.70	1.42	10	50.77	1.59	13
Clutch size	9.40	0.84	10	9.54	1.13	13
Brood size (at day 13)	8.40	2.07	10	8.69	1.18	13
Nestling level						
Hemoglobin (g/l)	129.57	14.50	30	119.65	11.58	37
Glucose (mg/dL)	233.33	37.60	30	251.08	39.89	36
Hematocrit (%)	45.55	3.85	30	43.34	3.44	35
Body mass (g)	16.45	0.89	30	16.15	1.68	37
Wing length (mm)	45.57	4.04	30	43.73	2.76	37

**Fig. 3.**
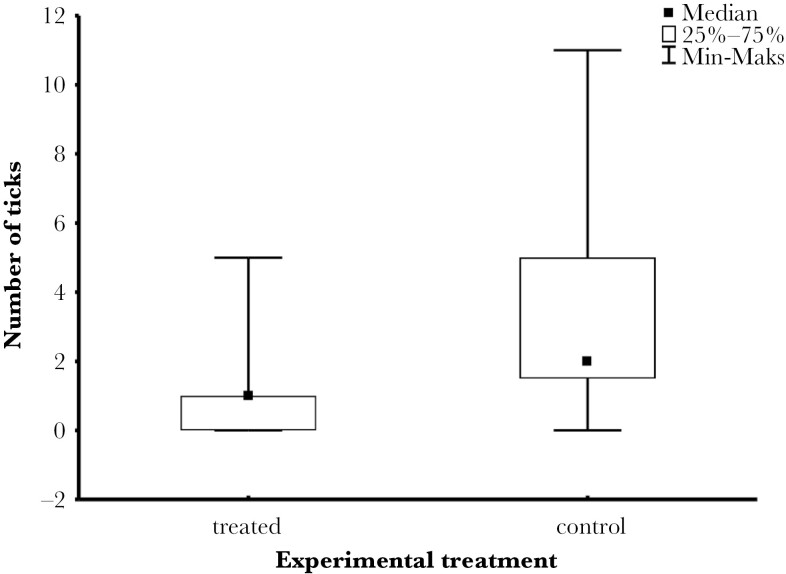
The number of ticks (Ixodidae) in aromatic plant experimentally—test and control groups.

No significant differences were found in total ectoparasite loads between the test and control groups. The most numerous ectoparasites were fleas (Ceratophyllidae) reaching very high numbers, with over 1,200 individuals observed in the sampled section. Fleas were represented mainly by larval stages feeding on detritus, but hematophagous imagines were also numerous in some nests reaching up to 220 individuals in a sample. Blow fly (Calliphoridae) larvae were rather uncommon and occurred in similar abundance (up to 3 larvae per sample) in test and control groups. Acari (Parasitiformes and Acariformes) were common, represented mainly by various blood-fed specimens of non-ixodid taxa, and did not differ between the control and test groups. However, hard ticks (Ixodidae) were significantly less abundant in the nests with aromatic plants added (*U* = 24.5, *P* < 0.03) (Fig. 3, Table 3).

**Table 3.** The abundance of nest-dwelling ectoparasitic arthropods between the 2 experimental groups of collected nests (treatment: control—0 and test—1).

Nest	Treatment	Siphonaptera	Diptera	*Protocalliphora*	Acari	Ixodida	Coleoptera	Dermestidae	Histeridae	Staphylinidae	Hymenoptera	Lepidoptera	Collembola	*Psocodea*	Hemiptera
**1**	1	1037	4	3	6	1	55	1	49	5	2	0	0	0	0
**2**	1	10	1	0	20	5	12	0	3	8	0	2	0	0	0
**3**	1	95	0	0	9	0	12	0	12	0	0	0	0	0	0
**4**	1	13	0	0	57	2	3	0	1	2	2	0	0	0	0
**5**	0	430	1	0	5	0	15	0	15	0	0	0	0	0	0
**6**	1	7	0	0	74	0	3	1	0	2	0	0	2	0	0
**7**	1	700	0	0	51	1	6	2	2	2	0	0	0	0	0
**8**	0	149	0	0	21	1	7	0	3	4	0	0	0	0	0
**9**	0	0	0	0	58	6	4	0	3	1	0	0	0	4	0
**10**	1	6	0	0	25	0	2	0	2	0	0	0	0	0	0
**11**	1	6	0	0	40	1	407	0	1	5	0	0	0	0	0
**12**	0	616	14	0	183	2	16	0	16	0	5	1	0	0	0
**13**	0	139	1	0	4	4	20	0	10	10	0	0	0	0	0
**14**	0	401	5	3	10	4	11	0	6	5	1	1	0	0	1
**15**	0	21	12	3	42	1	2	0	2	0	1	0	0	0	0
**16**	0	310	6	0	18	7	15	1	10	1	0	0	0	0	0
**17**	0	143	4	0	57	11	3	0	2	1	1	1	0	0	0
**18**	0	265	9	0	63	2	19	0	18	1	0	0	0	0	0
**19**	0	156	0	0	46	2	8	0	5	2	0	0	0	0	0
**20**	0	122	0	0	3	1	7	0	7	0	0	1	0	0	0
**21**	1	563	8	0	24	0	22	0	11	2	0	0	0	0	0
**22**	1	1238	2	2	194	0	4	0	1	2	0	0	0	0	0

## Discussion

In this study, we found that great tit nestlings in nests with experimentally added fresh aromatic plant fragments exhibited increased values for the multivariate blood indicator related to erythrocytes (PC1). This increase was driven by both higher hemoglobin levels (on average 9.9 g/l higher in the test group) and higher hematocrit levels (on average 2.3% higher in the test group) compared to control nests. However, there were no significant differences between the test and control groups in either body condition indicators (body mass and wing length), the glucose-dependent multivariate blood indicator (PC2), or glucose concentration.

We found that, compared to the control group, the test group had fewer parasites. Thus, the herbs demonstrated an anti-parasitic effect, placing this study among the few that report such findings (reviewed in Dubiec et al. 2013; Garrido-Bautista et al. 2023). Previous studies have yielded mixed results, showing either no discernible influence of fresh plant material on parasite loads (Fauth et al. 1991; Gwinner et al. 2000; Brouwer and Komdeur 2004; Gwinner and Berger 2005; Mennerat et al. 2008, 2009a), an effect in some populations but not in others (Clark and Mason 1988; but see Fauth et al. 1991), or an effect limited to specific parasite taxa (Dawson 2004; Shutler and Campbell 2007; Tomás et al. 2012). Our findings align with the latter, as we observed that the plant material only reduced the abundance of ticks, while having no significant effect on other nest ectoparasites, such as fleas, blow flies, and non-ixodid mites. It appears that some of the plants tested (or their combination) repelled ticks or affected their physiology but had no detectable impact on other ectoparasitic arthropods. Reports on the effect of plants in nests on different ectoparasite taxa remain inconclusive. For instance, the use of common yarrow *Achillea millefolium* had no effect in European starling nests (Gwinner et al. 2000), consistent with our results, but studies on tree swallows *Tachycineta bicolor* showed varying outcomes: a reduction in ceratophyllid flea loads in some cases (Shutler and Campbell 2007), and an increase in others (Dawson 2004). Lavender *Lavandula stoechas*, a species different from the one we used, was found to reduce flea numbers, but this effect was limited to the nests of yearling females (Tomás et al. 2012). Overall, the large body of research on the influence of fresh plant material on nest ectoparasites generally does not strongly support the nest protection hypothesis given that fresh aromatic plant fragments in bird nests had little effect on arthropod communities in most field studies.

In general, nest-dwelling ectoparasites may reduce future nestling survival and fitness (Richner et al. 1993; Merino and Potti 1995; Garrido-Bautista et al. 2022). For example, blowfly larvae *Protocalliphora azurea* are known to negatively affect the physiology and survival of tit nestlings (Tomás et al. 2007; Arriero et al. 2008), while fleas can impact nestling body mass and tarsus length (Richner et al. 1993; Merino and Potti 1995). However, these detrimental effects may only be noticeable in certain breeding seasons or populations due to the presence of other selective pressures, such as weather conditions or phenology. Glądalski et al. (2018a) replaced natural nests (twice, on the fifth and tenth days of the nestling stage) with artificial, sterile nests made from moss and cotton wool, which reduced parasitic pressure on great tit nestlings. In these nests, the nestlings showed higher hemoglobin concentrations than those in control nests, similar to the findings in our current experimental study. This suggests that the observed physiological differences between control and experimental nests in the present study may be attributed to the reduction of ectoparasite pressure rather than a direct effect of the plants on nestling condition, supporting the nest protection hypothesis. In another study, Glądalski et al. (2020) experimentally tested whether volatile compound-producing aromatic herbs in blue tit nests affected the physiological condition of nestlings, specifically regarding hemoglobin and glucose concentrations in the blood. Nestlings in nests with added aromatic plants also had elevated blood hemoglobin levels (mean 7.9 g/l), but there were no differences in glucose concentration. However, in that study, ectoparasite abundance was not evaluated. Our present and previous experimental studies on nest fumigation suggest that hemoglobin concentration is a reliable indicator of ectoparasite pressure (Słomczyński et al. 2006; Glądalski et al. 2018a, 2020). Some groups of blood-sucking ectoparasites have been shown to affect hematological indices in birds (Norte et al. 2013). Heylen and Matthysen (2008) demonstrated that ticks, as hematophagous ectoparasites with a broad range of terrestrial vertebrate hosts, can cause a decrease in hematocrit levels and an increase in the erythrocyte sedimentation rate in great tits. On the other hand, Garrido-Bautista et al. (2023) conducted a recent experimental study in which mint *Mentha* sp. was added to blue tit nests and found no effects of aromatic plants on nestling hemoglobin levels or any other physiological health metrics evaluated. Their correlative data also did not support the drug hypothesis. Overall, their results did not provide evidence that the use of aromatic plants affected the short-term survival of nestlings, despite being associated with a lower intensity of parasitism by fleas and obligatory parasitic mites. These contradictory results among studies may be attributed to varying parental abilities to compensate for the detrimental effects of parasites (Mennerat et al. 2009a). Mennerat et al. (2009a) reported positive effects of aromatic plants on the body condition (body mass and feather development) and hematocrit of blue tit nestlings. However, the effects on body condition were only noticeable in experimentally enlarged broods, whereas hematocrit was positively affected by the addition of aromatic plants regardless of brood size. Pires et al. (2020) found no effect of treatment on body condition in their experimental study with aromatic plants and blue tit nestlings, specifically regarding nestling weight and overall tarsus length. Similarly, Tomás et al. (2012) did not find any effects on hatching success, fledging success, or nestling body mass in their experiment with blue tits and aromatic herbs. Along with the present work, these studies suggest that some physiological indices of body condition (e.g. hematocrit and hemoglobin levels) may detect more subtle effects of parasitism than indices of body condition or fledging success. Although fledging success may not be affected (as in our study), a decreased anabolic capacity in nestlings due to reduced hemoglobin and hematocrit levels may still have repercussions during emancipation, winter survival, and recruitment (Minias 2015; Cornell et al. 2017; Brown et al. 2021).

## Data Availability

Analyses reported in this article can be reproduced using the data provided by Glądalski et al. (2024).
